# Anesthetic Management and Perioperative Outcome of Emergency Laparotomy in a Patient With Advanced Motor Neurone Disease: A Case Report

**DOI:** 10.1002/ccr3.73635

**Published:** 2026-09-28

**Authors:** Sherif Elmahgoub, Abdou Elshawa, Sean Flannery

**Affiliations:** ^1^ Galway University Hospital Galway Ireland

**Keywords:** amyotrophic lateral sclerosis, emergency laparotomy, genomic profiling, motor neurone disease, perioperative outcome, total intravenous anesthesia

## Abstract

Motor neurone disease (MND) presents significant perioperative challenges due to progressive respiratory muscle weakness, bulbar dysfunction, autonomic instability, and altered sensitivity to anesthetic agents. Emergency surgery further limits opportunities for optimisation and advance planning. We report the perioperative and anesthetic management of a patient with advanced MND undergoing emergency laparotomy for small bowel obstruction. A total intravenous anesthetic with cautious use of neuromuscular blockade was employed, and the patient was successfully extubated postoperatively. Despite an initially uncomplicated postoperative course, the patient subsequently developed progressive respiratory failure in the context of ongoing intra‐abdominal pathology. Following multidisciplinary discussion and shared decision‐making, care was transitioned to a comfort‐focused approach. This case highlights the limitations of perioperative intervention in advanced MND and emphasizes the importance of goal‐concordant care in emergency surgical settings.

## Key Clinical Message

Emergency anesthesia in patients with advanced motor neurone disease may be technically successful yet fail to alter overall prognosis. Early multidisciplinary discussions and realistic perioperative goal‐setting are essential to ensure patient‐centred care.

## Graphical abstract

Schematic summary of the perioperative management and clinical course of emergency laparotomy in a patient with advanced motor neurone disease (MND).

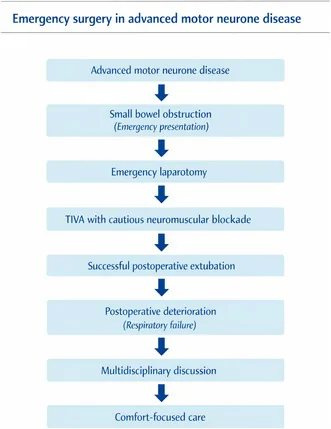

## 1 Introduction

Motor neurone disease (MND), also referred to as amyotrophic lateral sclerosis, is a progressive neurodegenerative disorder characterized by degeneration of upper and lower motor neurons. Respiratory muscle weakness, impaired cough, bulbar dysfunction, and autonomic instability significantly increase perioperative morbidity and mortality, with respiratory failure remaining the most common cause of death. Anesthetic management is further complicated by altered sensitivity to neuromuscular blocking agents and sedatives [1, 2, 3, 4].

While elective procedures allow for preoperative respiratory assessment and planning, emergency surgery presents unique challenges, often necessitating rapid decision‐making in the context of limited physiological reserve. We present a case of emergency laparotomy in a patient with advanced MND, focusing on anesthetic decision‐making, postoperative course, and ethical considerations.

## 2 Case History/Examination

A patient with established motor neurone disease presented with a 6–7‐day history of persistent vomiting, inability to tolerate oral intake, and progressive abdominal distension. Past medical history included MND, previous deep vein thrombosis and pulmonary embolism (managed with apixaban), diverticular disease, dyslipidaemia, and prior abdominal surgery (right hemicolectomy). Regular medications included riluzole and apixaban. There were no known drug allergies.

## 3 Methods (Differential Diagnosis, Investigations, and Treatment)

Computed tomography of the abdomen and pelvis demonstrated small bowel obstruction with a mid‐abdominal transition point, consistent with an internal hernia. Initial conservative management with nasogastric decompression, intravenous fluids, and antimicrobial therapy was unsuccessful, and the patient was scheduled for emergency laparotomy.

Given the underlying neuromuscular disease and aspiration risk, a modified rapid sequence induction was performed using total intravenous anesthesia with propofol and remifentanil target‐controlled infusions. Neuromuscular blockade was achieved using a reduced dose of rocuronium, administered cautiously due to concerns regarding increased sensitivity and prolonged blockade in patients with denervated muscle [2, 4]. Tracheal intubation was performed using video‐laryngoscopy. Invasive arterial monitoring and central venous access were established to facilitate haemodynamic management.

Intraoperatively, pressure‐controlled ventilation with volume guarantee was employed. Vasopressor support was required to maintain haemodynamic stability. A transversus abdominis plane block was performed to facilitate opioid‐sparing analgesia. The procedure proceeded without intraoperative complications.

## 4 Conclusion and Results (Outcome and Follow‐Up)

Following adequate reversal of neuromuscular blockade, the patient was successfully extubated and admitted to a high‐dependency unit for close postoperative monitoring. Analgesia was provided using low‐dose morphine patient‐controlled analgesia. The initial postoperative course was uncomplicated, and the patient was transferred to the ward the following day.

Subsequently, the patient developed worsening abdominal distension with large‐volume intra‐abdominal fluid collections requiring interventional radiology‐guided drainage and escalation of antimicrobial therapy. The clinical course was complicated by progressive hypoxaemia and Type 2 respiratory failure. Noninvasive ventilatory support was trialed but poorly tolerated.

In the context of advanced MND, limited physiological reserve, and previously established ceilings of care, a multidisciplinary discussion was held with the patient and family. A shared decision was made to transition to comfort‐focused care. The patient died peacefully shortly thereafter. Schematic summary of the perioperative course is illustrated in Figure 1.

**FIGURE 1:**
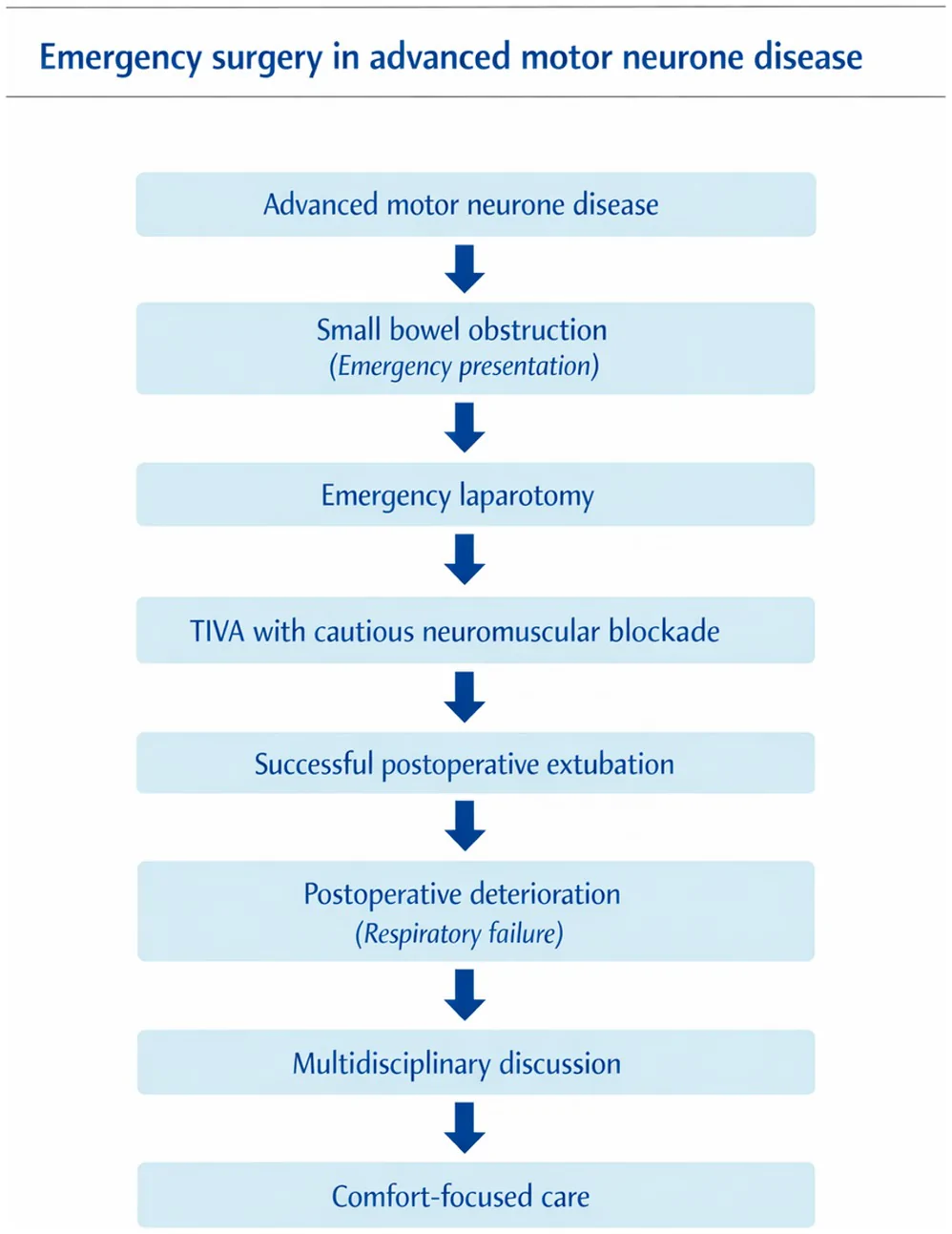
Schematic summary of the perioperative course.

## 5 Discussion

This case highlights the significant perioperative challenges associated with emergency surgery in patients with advanced MND. Progressive respiratory muscle weakness, impaired cough and secretion clearance markedly increase the risk of postoperative respiratory failure, even when immediate perioperative management is technically successful [1, 3, 5].

Total intravenous anesthesia was selected to allow precise titration of anesthetic depth and to avoid potential complications associated with volatile agents in neuromuscular disease [3, 4, 5, 6, 7]. Neuromuscular blocking agents were used cautiously due to the risk of exaggerated and prolonged effects related to denervation‐induced up‐regulation of extrajunctional acetylcholine receptors [2, 4]. Regional analgesia was employed to reduce opioid requirements and mitigate respiratory depression.

Beyond immediate perioperative pharmacodynamics, broader diagnostic modalities such as genetic and genomic profiling are increasingly recognized for their role in the comprehensive management of MND. In particular, whole‐blood transcriptome analysis and RNA profiling offer noninvasive insights into differential gene expression, molecular pathways, and disease activity [8]. Incorporating such transcriptomic signatures into standard clinical evaluation may in the future assist in predicting individual variations in drug metabolism and therapeutic response, thereby helping clinicians refine tailored anesthetic strategies and perioperative risk stratification [8].

Despite successful extubation and an initially stable postoperative course, the patient's subsequent deterioration underscores the limited capacity of perioperative interventions to modify disease trajectory in advanced MND. This case reinforces the importance of early multidisciplinary discussions, clear communication, and realistic goal setting to ensure care remains aligned with patient values, particularly in emergency surgical contexts.

## 6 Learning Points

- Advanced motor neurone disease confers a high risk of postoperative respiratory failure following emergency surgery.
- Total intravenous anesthesia with cautious use of neuromuscular blockade may reduce anesthetic‐related complications in MND.
- Initial perioperative success does not preclude subsequent clinical deterioration in advanced neuromuscular disease.
- Early multidisciplinary discussions and clearly defined ceilings of care are essential in emergency surgical settings.

## Author Contributions

**Sherif Elmahgoub:** conceptualization, methodology, project administration, supervision, writing – original draft, writing – review and editing. **Abdou Elshawa:** investigation, resources, visualization. **Sean Flannery:** data curation, investigation, writing – original draft.

## Funding

The authors have nothing to report.

## Ethics Statement

The authors have nothing to report.

## Consent

Written informed consent was obtained from the patient's next of kin for publication of this case report.

## Conflicts of Interest

The authors declare no conflicts of interest.

## Data Availability

Data sharing not applicable – no new data generated, or the article describes entirely theoretical research.

## References

[1] D. Paul , M. Wright , J. Palmer , and T. Russell , “Perioperative Management of Patients With Amyotrophic Lateral Sclerosis: A Narrative Review,” Anaesthesia and Intensive Care 50, no. 5 (2022): 345–360. 10.1177/0310057X211065042 36076353

[2] A. Romero and G. P. Joshi , “Neuromuscular Disease and Anesthesia,” Muscle & Nerve 48, no. 3 (2013): 451–460. 10.1002/mus.23817 23424048

[3] F. Racca , T. Mongini , A. Wolfler , et al., “Recommendations for Anesthesia and Perioperative Management of Patients With Neuromuscular Disorders,” Minerva Anestesiologica 79, no. 4 (2013): 419–433. 23419334

[4] J. A. Katz and G. S. Murphy , “Anesthetic Considerations for Neuromuscular Diseases,” Current Opinion in Anaesthesiology 30, no. 3 (2017): 435–440. 10.1097/ACO.0000000000000466 28448298

[5] L. R. Van den Bersselaar , M. Gubbels , S. Riazi , et al., “Mapping the Current Evidence on the Anesthetic Management of Adult Patients With Neuromuscular Disorders: A Scoping Review,” Canadian Journal of Anaesthesia 69, no. 6 (2022): 756–773. 10.1007/s12630-022-02230-3 PMC9132812 35322378

[6] L. R. Van den Bersselaar , L. Heytens , H. C. A. Silva , et al., “European Neuromuscular Centre Consensus Statement on Anaesthesia in Patients With Neuromuscular Disorders,” European Journal of Neurology 29, no. 12 (2022): 3486–3507. 10.1111/ene.15526 PMC9826444 35971866

[7] G. J. L. Van Neerven , W. J. Schelling , K. van den Borne , et al., “The Periprocedural Respiratory Safety of Propofol Sedation in Patients With a Motor Neuron Disease Undergoing Percutaneous Endoscopic Gastrostomy Insertion,” Journal of the Neurological Sciences 461 (2024): 123049. 10.1016/j.jns.2024.123049 38788288

[8] S. Kõks , K. Rallmann , M. Muldmaa , et al., “Whole Blood Transcriptome Profile Identifies Motor Neurone Disease RNA Biomarker Signatures,” Experimental Biology and Medicine 249 (2024): 10401, 10.3389/ebm.2024.10401. PMC11750576 39844875

